# Pregnancy after Prosthetic Aortic Valve Replacement: How Do We Monitor Prosthetic Valvular Function during Pregnancy?

**DOI:** 10.1155/2018/4935957

**Published:** 2018-01-17

**Authors:** Nicole Sahasrabudhe, Nickolas Teigen, Diana S. Wolfe, Cynthia Taub

**Affiliations:** ^1^Department of Obstetrics and Gynecology, Albert Einstein College of Medicine, Bronx, NY, USA; ^2^Department of Cardiology, Albert Einstein College of Medicine, Bronx, NY, USA

## Abstract

**Background:**

With modern medicine, many women after structural heart repair are deciding to experience pregnancy. There is a need for further study to identify normal echocardiographic parameters to better assess prosthetic valvular function in pregnancy. In addition, a multidisciplinary approach is essential in managing pregnant patients with complex cardiac conditions.

**Case:**

A 22-year-old nulliparous woman with an aortic valve replacement 18 months prior to her pregnancy presented to prenatal care at 20-week gestation. During her prenatal care, serial echocardiography showed a significant increase in the mean gradient across the prosthetic aortic valve. Multidisciplinary management and a serial echocardiography played an integral role in her care that resulted in a successful spontaneous vaginal delivery without complications.

**Conclusion:**

Further characterization of the normal echocardiographic parameters in pregnant patients with prosthetic valves is critical to optimize prenatal care for this patient population. This case report is novel in that serial echocardiograms were obtained throughout prenatal care, which showed significant changes across the prosthetic aortic valve.

**Teaching Points:**

(1) Further study is needed to identify normal echocardiographic parameters to best assess prosthetic valvular function in pregnancy. (2) Multidisciplinary management is encouraged to optimize prenatal care for women with prosthetic aortic valve replacements.

## 1. Introduction

Normal pregnancy induces major hemodynamic changes that require significant cardiac adaptations [1]. Increases in heart rate and plasma volume are associated with a dramatic increase in cardiac output [2]. In addition, there is a decrease in afterload due to a decrease in systemic vascular resistance during pregnancy. With modern medical and surgical techniques, many women with congenital heart disease or acquired cardiac disease are now finding themselves with improved survival and quality of life after surgical repair and are choosing to experience pregnancy.

Currently, one of the most common types of heart disease encountered during pregnancy is congenital heart disease after structural heart repair [3]. Studies have shown that hemodynamic changes of pregnancy increase the risk of cardiac complications for patients with valvular repair or replacements. In 2014, the American Heart Association and American College of Cardiology guidelines for the management of patients with valvular heart disease acknowledged that, due to an increase in cardiac output that occurs during pregnancy, the mean pressure gradient across all prostheses will increase throughout gestation [4]. The guidelines encourage the use of other hemodynamic parameters such as dimensionless index to determine the function of the aortic prosthesis. However, it does not clarify what normal echocardiographic parameters are to compensate for these hemodynamic changes in pregnancy. The European Society guidelines highlight the importance of anticoagulation during pregnancy but provide no clear guidelines as to how to monitor prosthetic valvular function in pregnancy [5].

Literature regarding management and prenatal outcomes of patients with prosthetic valves is scarce [6]. Literature on echocardiographic changes in pregnant patients with prosthetic valves is even more limited [7]. In our case report, we present a woman with a bioprosthetic aortic valve with a successful pregnancy course through the management of our MFM-Cardiology Joint program.

## 2. Case

A 20-year-old female presented to the emergency department with chest pain, dyspnea, fever, and chills for 3 weeks. She was an active intravenous heroin abuser at the time and had been attempting to wean herself off heroin. During her evaluation, her blood cultures were notable for* Streptococcus mitis*. The diagnosis of bacterial endocarditis with severe aortic valve vegetation was made after a transthoracic echocardiography was performed. The patient was started on intravenous antibiotics on the day of admission and a cardiothoracic surgery consultation was made. On hospital day number 2, the patient was taken to the operating room for a bovine pericardial patch repair of a fistula and aortic valve replacement with a 21 mm bioprosthetic valve. The patient remained hospitalized for 36 days and was discharged to a drug rehabilitation program once she was medically cleared.

When the patient presented to cardiology for follow-up 1.5 years after her aortic valve replacement, the patient was 22 years old and 20 weeks pregnant with a recorded body mass index of 29 kg/m^2^. The patient had been enrolled in a methadone maintenance program and had not used intravenous heroin for 6 months. She was promptly referred to our maternal-fetal medicine and cardiology joint program, a multidisciplinary collaboration created to manage pregnant women with preexisting or acquired cardiac conditions. An echocardiogram was performed during her first prenatal care visit showing a heart rate of 61 bpm, peak velocity of 360.5 m/s, peak gradient across valve of 52 mmHg, mean gradient across valve of 28.9 mmHg, and dimensionless index 0.36.

Joint prenatal care and cardiology clinic visits were scheduled, during which routine prenatal care and cardiology evaluations were performed. The patient was placed on 81 mg of aspirin for the duration of her pregnancy to decrease her risk of thrombosis. Echocardiogram was repeated at 32 weeks and 37 weeks of gestation. At 32-week gestation, the echocardiogram showed a heart rate of 69 bpm, peak velocity of 386.9 m/s, peak gradient across valve 59.9 mmHg, mean gradient across valve 36.1 mmHg, and dimensionless index 0.43. At 37-week gestation, heart rate was 76, peak velocity of 339.3 m/s, peak gradient across valve 46.1 mmHg, mean gradient across valve 22.6 mmHg, and dimensionless index 0.45. The patient was asymptomatic and denied any cardiac symptoms.

A multidisciplinary meeting included subspecialists of cardiology, maternal-fetal medicine, anesthesiology, critical-care, and labor and delivery nursing to create a delivery plan. A consensus was reached that a vaginal delivery would be safe for this patient without an assisted second stage, unless obstetrically indicated. A plan was made for telemetry monitoring immediately postpartum given higher risk of arrhythmia at that time.

The patient presented to the labor and delivery triage unit at 40-week 0-day gestation complaining of contractions and was found to be in spontaneous labor. As discussed during our multidisciplinary meeting, the patient received endocarditis prophylaxis and underwent expectant management of her labor. The patient did not require an assisted second stage of delivery and proceeded to have a spontaneous vaginal delivery. Within 10 hours of admission, she delivered a healthy male infant with Apgar scores of 9 at 1 min and 9 at 5 min, weighing 3770 grams with estimated blood loss of 350 ml. Her postpartum care was uneventful. Her echocardiography on postpartum day 1 showed a heart rate of 63 bpm, peak velocity of 325.8 m/s, peak gradient across valve 42.5 mmHg, mean gradient across valve 25.3 mmHg, and dimensionless index 0.36. The patient was discharged on postpartum day 3 without any complications. At her 6-week postpartum visit, the patient received an intrauterine device (IUD) for contraception.

## 3. Discussion

The assessment and management of prosthetic heart valves during pregnancy pose several clinical challenges. Data on prosthetic aortic valve function in pregnancy is limited and high transaortic gradients observed during pregnancy may be concerning. Therefore, it is imperative that a multidisciplinary approach be used to optimize pregnancy outcomes.

Pregnancies in women with prosthetic heart valves have been associated with an increased incidence of adverse outcomes. Lawley et al., in a meta-analysis of 11 studies capturing 499 pregnancies among women with heart valve prosthesis, pooled estimate of maternal mortality was 1.2/100 pregnancies, for mechanical valves subgroup 1.8/100 and bioprosthetic subgroup 0.7/100, overall pregnancy loss 20.8/100 pregnancies, perinatal mortality 5.0/100 births, and thromboembolism 9.3/100 pregnancies [8]. Despite these risks, the hemodynamic adaptations of pregnancy may be well tolerated in women with bioprosthetic valves as long as the valve is functioning normally, and there is no other significant cardiac disease. However, there still is limited data to illustrate optimal monitoring guidelines for pregnant patients with prosthetic heart valves. Additionally, the normal echocardiographic parameters for these prosthetic heart valves to accommodate the hemodynamic changes of pregnancy are not well understood.

Data on hemodynamic adaptations in pregnancy in women with structural heart disease are scarce and have not been described in a longitudinal manner [9]. The 2014 American Heart Association and American College of Cardiology Guidelines for patients with valvular heart disease acknowledge that the mean pressure gradients across all prostheses will increase throughout gestation secondary to an increase in cardiac output [4]. The guidelines also encourage the use of other hemodynamic parameters such as dimensionless index to determine the function of the aortic prosthesis. The European Society guidelines also highlight the importance of anticoagulation during pregnancy for these patients but no clear recommendations exist for monitoring prosthetic valvular function in pregnancy such as interval echocardiogram and normal echocardiographic parameters with the changes in pregnancy [5].

However, there is no concrete guideline as to how obstetricians and cardiologists can optimally manage these patients other than getting a baseline and repeat echocardiograms if the patient becomes symptomatic. There has been little published regarding normal and abnormal echocardiography parameters in pregnant patients with prosthetic valves. Normal and abnormal echocardiographic parameters have been described for nonpregnant patients with prosthetic aortic valves, but there is limited data as to the changes in these parameters due to hemodynamic changes of pregnancy [10].

**Figure 1:**
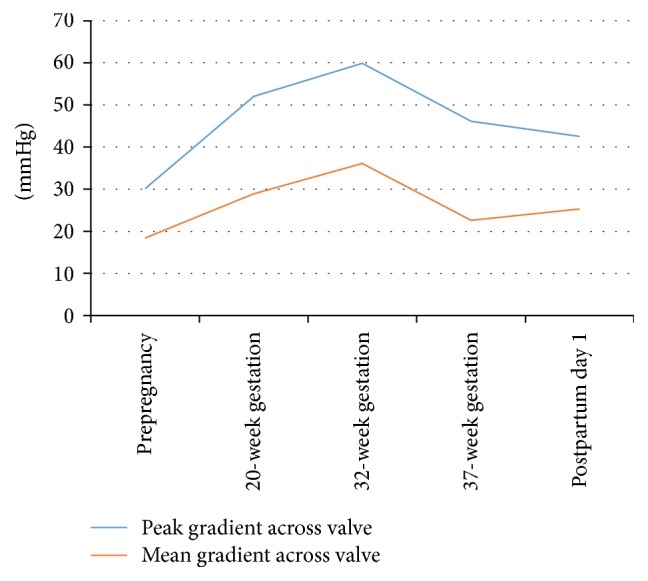
Serial echocardiographic measurements of the mean and peak prosthetic aortic valve gradients over time.

**Figure 2:**
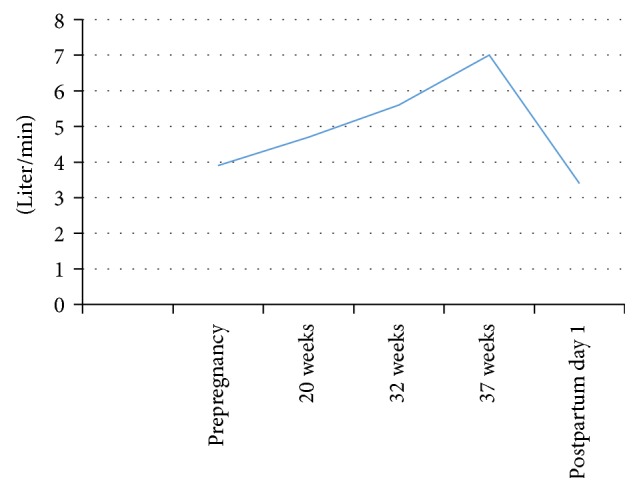
Changes in cardiac output during prepregnancy, antepartum, and postpartum periods.

In our patient, there was a significant increase in the mean gradient across the prosthetic aortic valve until around 32 weeks to 36.1 mmHg (Table 1), which could be interpreted as significant aortic prosthetic valve stenosis in nonpregnant patients. But it is difficult to interpret the data as such, given that we do not have enough information to identify stenosis in the pregnant population. Hemodynamic changes of pregnancy with a substantial increase in cardiac output were likely responsible for this change, especially since our patient did not experience signs and symptoms of aortic stenosis. Throughout her pregnancy, we observed a gradual increase in the mean gradient across the bioprosthetic aortic valve until around 32 weeks and subsequent stabilization during the rest of third trimester and return to baseline soon after the delivery (Figure 1). The same trend is observed for cardiac output throughout her pregnancy (Figure 2).

Lesniak-Sobelga and colleagues studied pregnancy outcomes of 259 women with cardiac disease, of which 54 patients had aortic valve disease [6]. This study reports that, in women with severe aortic stenosis, pregnancy can lead to sudden clinical deterioration. Results of echocardiographic examinations revealed an increase in aortic gradients throughout gestation. However, these were patients with native valve dysfunctions.

Heuvelman et al. described pregnancy outcomes of 40 women after successful aortic valve replacements [7]. There were increased maternal cardiac and obstetrical complications such as heart failure, arrhythmia, valve thrombosis, preeclampsia, and preterm delivery. The authors recommend careful monitoring of these high risk patients but there are no concrete guidelines as to how that can be accomplished.

Limited data suggests that, for patients with bioprosthetic valves, pregnancy may accelerate structural valve degeneration [10]. The team caring for a pregnant woman with history of aortic valve replacement or any valvular dysfunction should be prepared for possible maternal cardiac decompensation. Creating a multidisciplinary delivery plan with maternal-fetal medicine, cardiology, anesthesia, and labor and delivery nursing plays a critical role in successful perinatal outcomes.

This case describes the use of multidisciplinary planning and serial echocardiography throughout pregnancy as a way to monitor and improve perinatal outcomes. In the past, pregnant patients with aortic valve disease posed a great risk of maternal mortality and morbidity. Now with modern medicine, these patients are undergoing successful aortic valve replacements with improved quality of life and are choosing to experience pregnancy. However, there is limited information on how to best assess and evaluate pregnant patients with prosthetic valves. This case report is novel in that serial echocardiograms were obtained throughout prenatal care, which showed significant changes across the prosthetic aortic valve. The significance of these changes is not well understood. Therefore, there is a need for further data in obtaining serial echocardiograms on pregnant patients with prosthetic valves to delineate what normal echocardiographic parameters are in order to assess their cardiac risk during pregnancy. Because the physiologic changes of pregnancy can have an impact on prosthetic valvular function, we recognize that it is crucial to develop clear echocardiographic parameters to stratify their risk of cardiac complications.

Our case also illustrates that patients with serious medical comorbidities such as our patient with aortic valve replacement may greatly benefit from a multidisciplinary team approach to optimize their medical care.

## Figures and Tables

**Table 1:** Serial echocardiogram measurements through gestation.

Echocardiographic parameters						
Gestational age	Heart rate (bpm)	Peak velocity (cm/sec)	Peak gradient across valve (mmHg)	Mean gradient across valve (mmHg)	Pulse wave velocity time integral (m)	Dimensionless index
Prepregnancy	63	274.2	30.1	18.4		
20 weeks	61	360.5	52	28.9	31.2	0.36
32 weeks	69	386.9	59.9	36.1	35.7	0.43
37 weeks	76	339.3	46.1	22.6	29.2	0.45
Postpartum day 1	63	325.8	42.5	25.3	26.7	0.36
